# Impact of an Injectable Trace Mineral Supplement on the Immune Response and Outcome of *Mannheimia haemolytica* Infection in Feedlot Cattle

**DOI:** 10.1007/s12011-024-04251-z

**Published:** 2024-06-10

**Authors:** Suyeon Hong, Emma L. Rients, Carlos E. Franco, Stephanie L. Hansen, Jodi L. McGill

**Affiliations:** 1 Department of Veterinary Microbiology and Preventive Medicine, Iowa State University, 1907 ISU C-Drive, Ames, IA 50011 USA; 2 Department of Animal Science, Iowa State University, Ames, IA 50011 USA

**Keywords:** Bovine respiratory disease, Injectable trace mineral, Immune function, Feedlot cattle

## Abstract

The study aimed to assess the impact of injectable trace mineral (“ITM”; Multimin90; Fort Collins, CO) supplementation on bacterial infection in cattle. Angus-crossbred steers (*n* = 32) were organized into two blocks by initial body weight. Steers were maintained on a ryelage and dry-rolled corn-based growing diet without supplementation of Zn, Cu, Mn, and Se for the duration of the study. The steers were transported 6 h, then randomized into three treatment groups: control received sterile saline (“CON”), ITM administered 1 day after transport (6 days before infection, “ITMPRE”), and ITM administered 2 days post infection (dpi) concurrent with antibiotic treatment (“ITMPOST”). Steers were infected with *Mannheimia haemolytica* on day 0, and all were treated with tulathromycin at 2 dpi. Plasma levels of Zn, Cu, and Se did not differ among treatments (*P* ≥ 0.74). Liver Se was higher in ITMPRE at 2 dpi (*P* < 0.05), and both ITM groups had higher liver Se at 5 dpi (*P* < 0.05) compared to CON. A time × treatment interaction was detected for liver Cu (*P* = 0.02). Clinical scores were lower (*P* < 0.05) in ITMPRE on 1 and 8 dpi and ITMPOST on 8 dpi compared to CON. Thoracic ultrasonography scores were lower in ITMPRE at 2 dpi compared to CON (*P* < 0.05) and ITMPOST (*P* < 0.1). No treatment effects (*P* > 0.10) were observed for bacterial detection from bronchoalveolar lavage (BAL) or nasopharyngeal swabs. At 5 dpi, both ITMPRE and ITMPOST showed higher frequencies of γδ T cells and NK cells in BAL compared to CON (*P* < 0.05). Before infection, leukocytes from ITMPRE steers produced more IL-6 (*P* < 0.01) in response to stimulation with the TLR agonist, Pam3CSK4. Use of ITM may be an effective strategy for improving disease resistance in feedlot cattle facing health challenges.

## Introduction

Bovine respiratory disease (BRD) is a leading cause of morbidity, mortality, and production losses among U.S. beef cattle. *Mannheimia haemolytica* (*M. haemolytica*) is the most common bacterial agent isolated in cases of BRD in feedlot cattle [1]. *M. haemolytica* is commonly found as a commensal bacterium in the upper respiratory tract of cattle. However, stress, viral infection, or suppression of the host immune response can lead to overgrowth of the organism in the upper respiratory tract and eventual bronchopneumonia and infection in the lower respiratory tract [2].

Trace minerals (TM), such as selenium (Se), zinc (Zn), copper (Cu), and manganese (Mn) play an important role in supporting the immune system of beef cattle. Numerous studies have shown positive effects of TM supplementation on immune function such as promoting neutrophil migration and phagocytic function, antibody production, and lymphocyte proliferative responses [3–7]. Supplementation with TM is particularly important when an animal is at increased risk for disease. As such, it is common practice to use high doses of oral TM supplements in newly received or at-risk calves [8, 9]. However, feed intake in receiving calves can be inconsistent, and the bioavailability of oral TM supplements varies widely among animals [9–12]. Injectable TM (ITM) offers an additional option to support the TM needs and immune system. Several studies have shown that ITM administration concurrent with vaccination can enhance humoral immune responses in both dairy and beef cattle [5, 6]. In one study, administration of ITM to newly received beef calves at the same time as a parenteral modified live vaccine induced more potent and rapid antibody responses against bovine viral diarrhea virus (BVDV) 1 and 2 [13]. When calves were challenged with BVDV 5 days later, animals receiving the ITM at the same time as the MLV experienced better health scores and a smaller decrease in platelets and leukocytes as a result of the BVDV challenge compared to controls [13]. In another study, ITM treatment concurrent with an intranasal vaccine, followed by a BVDV and bovine herpesvirus 1 (BHV-1) challenge resulted in reduced inflammatory responses and virus-associated pathology in the respiratory tract [14].

Despite the benefits of ITM administration at the time of vaccination, administration of ITM in morbid animals has not been well explored. Acute illness and inflammation lead to a sharp decrease in the concentrations of several circulating TM, a process often termed nutritional immunity [15]. This restricts the availability of TM to pathogens, but also reduces availability of essential TM to the functioning immune system. In receiving cattle, there are two most likely times for administration of ITM: during processing at receiving or when a calf is suspected of BRD and pulled for treatment. It is not known if administration of ITM is appropriate for the morbid animal or how this would impact the disease process and developing immune response. Therefore, the objective of the current study was to determine the effect of ITM administration (Se, Zn, Cu, and Mn) on cattle before or during treatment for a bacterial infection. We hypothesized that ITM administration both prior to and during treatment for a bacterial infection would support the immune response and improve the outcome of a *M. haemolytica* infection in receiving calves.

## Materials and Methods

The Iowa State University Institutional Animal Care and Use Committee approved this study’s procedures and protocols (IACUC-22-137). The study was performed at the Iowa State University Beef Nutrition Farm (BNF) (Ames, IA) and Animal Resource Station (ARS) (Ames, IA) from October through December 2022.

### Animals and Experimental Design

**Fig. 1:**
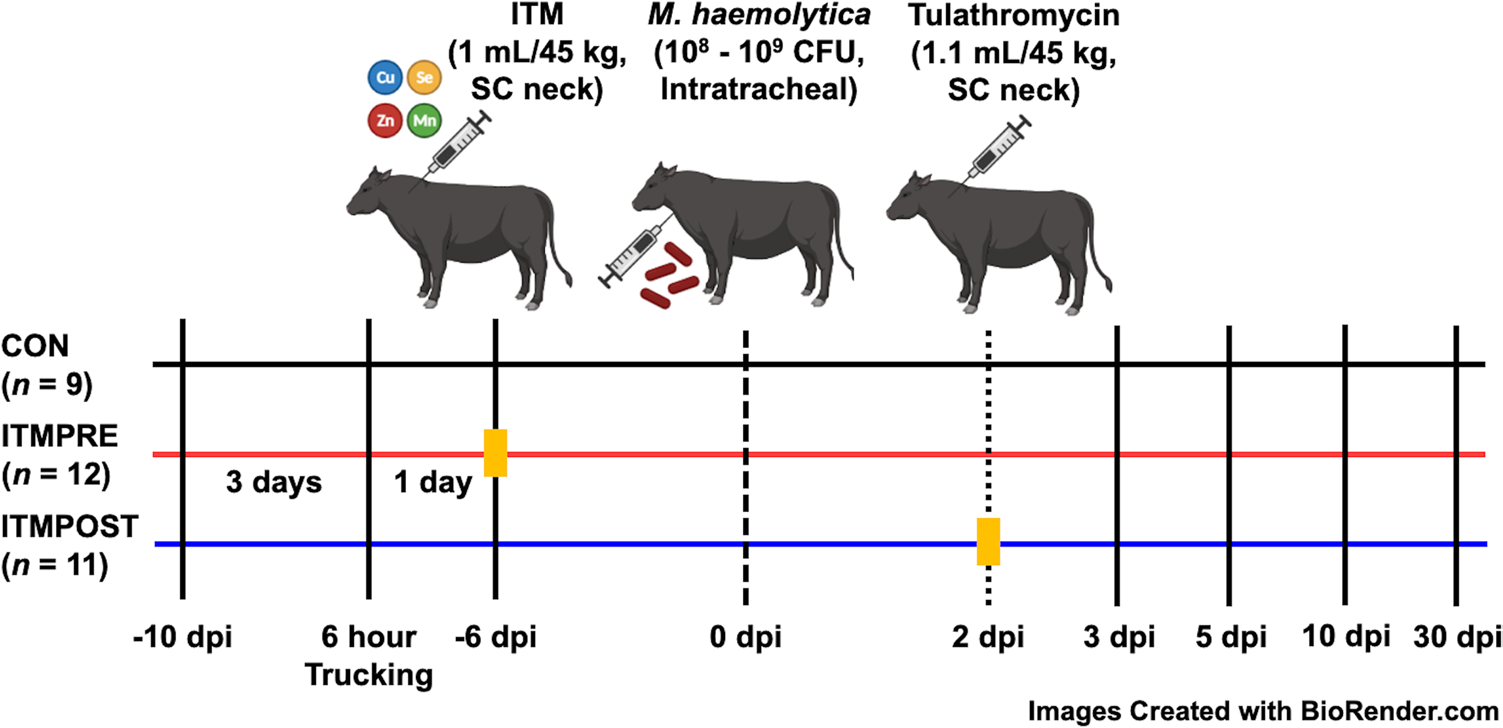
Experimental timeline of the injectable trace mineral (ITM) treatment, *M. haemolytica* infection, and antibiotic injection. A baseline liver biopsy sample was collected from all animals (*n* = 32) at −10 days post infection (dpi), which was 3 days prior to transport. Steers were then transported for 6 h to dampen the immune system. ITM-treated groups received subcutaneous (SC) ITM (1 mL/45 kg of BW, which included Cu = 15 mg/mL, Se = 5 mg/mL, Mn = 10 mg/mL, Zn = 60 mg/mL, yellow squares) injections in the neck. ITMPRE received ITM on day 1 post trucking (6 days before *M. haemolytica* infection (*n* = 12)). ITMPOST received ITM concurrently with an antibiotic at 2 dpi of *M. haemolytica* (*n* = 11). CON received sterile saline (1 mL/45 kg of BW) instead of ITM as control (*n* = 9). Steers were infected with *M. haemolytica* at 7 days post trucking, followed by antibiotic (tulathromycin) treatment at 2 dpi of *M. haemolytica*. All were challenged via intratracheal inoculation with *M. haemolytica* strain D153 at 9.48 × 10^8^ CFU per animal. All received tulathromycin (1.1 mL/45 kg of BW) at 2 dpi. All sample collections were performed before beginning ITM treatment, *M. haemolytica* infection, and antibiotic treatment. Images created with BioRender.com.

Thirty-six Angus-cross steers were purchased from a single source farm located in southern Iowa. Prior to disease challenge, steers were transported to the BNF and housed for 4 or 6 weeks for blocks 1 and 2, respectively. Steers were weighed, given a new EID and initially sorted by body weights (BW) into potential challenge groups 3 days after arrival. Thirty-six Angus-cross steers received a ryelage and dry-rolled corn-based growing diet (Table 1) without supplementation of Zn, Cu, Mn, and Se, fed ad libitum throughout the study. The diet was fed once daily at approximately 0800 h via a wagon mixer. According to the source farm, the steers did not receive any vaccinations prior to purchase or enrollment in the study. Cattle were monitored daily for signs of illness prior to disease challenge and excluded from the study if they were treated with antibiotics within 2 weeks of bacterial infection. Steers were blocked by BW (block 1, *n* = 18, BW = 282.9 $\pm$ 2.0 kg; block 2, *n* = 18, BW = 284.3 $\pm$ 2.3 kg) on 2 consecutive days before trucking. The study was performed in two blocks to accommodate pen space and sampling logistics. Blocks were separated by 3 weeks with identical feeding, treatment, and sampling logistics to accommodate replicating experiments. Four steers from block 2 were removed from the block because they developed foot rot and required antibiotic treatment. The final enrollment consisted of 32 Angus-crossbred steers randomly stratified by initial BW (block 1, *n* = 18, BW = 282.9 $\pm$ 2.0 kg; block 2, *n* = 14, BW = 284.2 $\pm$ 2.3 kg). Steers were trucked for 6 h to dampen the immune system and delivered to ARS. Animals were then divided into one of three treatment groups: (1) “CON,” sterile saline (1.1 mL/45 kg of BW) injection as control (*n* = 9); (2) “ITMPRE,” administration of injectable trace mineral (“ITM”: Multimin90; MULTIMIN90^®^ USA Inc., Fort Collins, CO) at a dose of 1 mL/45 kg of BW on day 1 post trucking (6 days before *M. haemolytica* infection) (*n* = 12); (3) “ITMPOST,” administration of ITM (1 mL/45 kg of BW) concurrent with an antibiotic at 2 days post *M. haemolytica* infection (*n* = 11) (Fig. 1). For treatment groups, either saline or ITM was injected on the neck subcutaneously. ITM contains 15 mg/mL of Cu, 5 mg/mL of Se, 10 mg/mL of Mn, and 60 mg/mL of Zn. All animals were intratracheally infected with *M. haemolytica* strain D153 at 9.48 $\times$ 10^8^ colony forming units (CFU) per animal at 7 days post trucking. All received tulathromycin (Draxxin, Zoetis) (1.1 mL/45 kg of BW) at 2 days post infection (dpi). All sample collections were conducted before commencing ITM treatment, *M. haemolytica* infection, and antibiotic treatment.

**Table 1:** Composition of diets fed

%DM		51.82
Ingredient, % DM basis		
	Ryelage	20
	Sweet Bran	35
	Corn	20
	Dried distillers grains^1^	23.08
	Limestone	1.5
	Vitamin A and E premix^2^	0.1
	Salt	0.31
	Rumensin	0.0135
	Trace mineral premix^3^	0.0007
Analyzed composition^4^, % DM		
	Crude protein	23.41
	Neutral detergent fiber	31.33
	Ether extract	6.29
Analyzed^5^, mg/kg of diet DM		
	Cu	5
	Zn	40.5
	Mn	19.9
	Se	0.31

^1^Carrier for microingredients

^2^Premix provided 2200 IU vitamin A and 25 IU vitamin E/kg diet DM

^3^Provided per kilogram of diet DM: 0.15 mg of Co and 0.5 mg of I

^4^Based on analysis of TMR from Dairyland Laboratories, Inc., Arcadia, WI

^5^Diet was unsupplemented. Analyzed mineral values reflect diet total and were determined by Iowa State University Veterinary Diagnostic Lab (Ames, IA)

### Clinical Score Assessment

Clinical examination was performed once daily from day −1 through 9 dpi by a single trained observer. Cattle were assigned a daily clinical score using the DART system (depression, appetite, respiratory changes, and temperature) [16]. Rectal temperatures were collected whenever the animal was handled in the chute (on −6, 0, 2, 3, 5, and 10 dpi).

### Thoracic Ultrasonography

Thoracic ultrasonography (TUS) was performed to monitor lung consolidation in response to *M. haemolytica* infection. TUS was assessed on days 0, 2, 3, 5, 10, and 30 with the *M. haemolytica*-challenged cattle. TUS was performed with an IBEX® EVO® (E.I. Medical Imaging, Loveland, CO) using the L7HD linear transducer probe (5 to 9 MHz) set to a depth of 11.6 cm (for cattle). The thorax was shaved on day 0 of the study, and 70% ethanol was applied to the thorax to enable TUS reading [17]. The ultrasound locations (intercostal space (ICS)) and scoring system were modified from [17–19] with the addition of one extra ICS, the right side (ICS 23 Right) on the lungs to capture a wider and more in-depth look into the state of the lungs during infection. A total of five locations on the left side (ICS 19 L, 18 L, 14 L, 10 L, and 9 L) and five on the right side (ICS 23R, 22R, 18R, 19R, and 14R) were scored for TUS. Scoring for TUS was modified from Rademacher et al. (2013) and has been previously reported (Porter et al. 2021). In brief, fewer than five pleural defects (comet tails) yields a score of 0; five or more pleural defects but no consolidation is scored as a 1. Lung consolidation receives a score of 2, 3, or 4 that is dependent upon the maximum depth of consolidation, with a score of 1 ≤ 2 cm of consolidation, 2 ≥ 2 cm but < 4 cm of consolidation, and 3 ≥ 4 cm of consolidation.

### *Mannheimia haemolytica* Infection

Cattle were infected with *M. haemolytica* strain D153 7 days after trucking (day 0, Fig. 1). *M. haemolytica* was prepared as previously described [20]. Briefly, pure cultures of *M. haemolytica* were grown on blood agar plates in a non-gassed incubator at 37 ℃ at 2 days prior to challenge. Six hours prior to challenge, isolated colonies were suspended in brain-heart infusion (BHI) broth and grown in 37 ℃ shaking incubator. After confirming optical density (OD) at 600 nm based on a *M. haemolytica* growth curve, the bacteria in log phase were pelleted and washed two times with sterile PBS at 3000 x g for 10 min, then resuspended in sterile saline. The dose of *M. haemolytica* was confirmed by quantitative culture on BHI agar. Cattles were challenged via intratracheal inoculation with 9.48 $\times$ 10^8^ colony forming units per animal (*n* = 32) *M. haemolytica* strain D153. *M. haemolytica* was suspended in 60 mL of sterile saline, followed by a 60 mL saline to wash the solution into the lungs.

### Blood Collection

Blood was collected via the jugular vein at 0, 2, 5, and 10 dpi into marble-top serum separation tubes for haptoglobin and C-reactive protein ELISA assays, K2 EDTA tubes for evaluation of plasma trace mineral concentration, and sodium heparin tubes for cell based immune assays. Serum and plasma tubes were centrifuged at 2000xg for 20 min and aliquoted to be stored at −80 ℃ for future analysis. Plasma samples were submitted to the Veterinary Diagnostic Laboratory at Iowa State University, where inductively coupled plasma mass spectrometry (ICP-MS) was used to determine TM concentrations.

### Bronchoalveolar Lavage Fluid Collection

Bronchoalveolar lavage (BAL) fluid was collected from all animals at 0, 2, 5, and 10 dpi using a protocol we have previously described with minor modifications [21]. A silicone, large-animal BAL catheter (10 mm x 2.5 mm x 240 cm long, MILA International) was passed through the nose and the trachea until reaching the bronchus. A total of 180 mL of sterile saline was divided into three aliquots. An aliquot was introduced using 60 mL syringes to the lower respiratory tract, and 30 mL of air was added, followed by immediate suction to obtain lower airway washes. The procedure was repeated twice more. All BAL fluids (BALF) were pooled, kept on ice, and filtered over sterile gauze. BALF were centrifuged at 2000 x g for 10 min. Cells were washed with cold PBS, counted, and stimulated as described below for ex vivo assays for ELISA. For bacterial recovery, filtered BALF was stored in glycerol (24% final concentration) to preserve bacteria until culturing on blood agar plates.

### Liver Biopsy

Liver biopsies were collected from all animals on days −10, 2, and 10 after *M. haemolytica* challenge. Liver biopsies were collected as described [22]. Biopsy samples were stored on ice until transport to the lab, then stored at −20 ℃. Liver samples were submitted to the Iowa State University Veterinary Diagnostic Laboratory for TM quantification by ICP-MS.

### Bacterial Recovery from BAL

BAL stored in glycerol (24% final concentration) was thawed and centrifuged at 3000 x g for 10 min for pelleting bacteria. Pellets were resuspended in 100 µL saline and plated on blood agar plates. After 2 days plating, colonies were evaluated quantitatively for *M. haemolytica* growth.

### Bacterial Genomic DNA Isolation and Quantitative Real-time PCR (qPCR)

Nasopharyngeal swab (NPS) samples were collected at 0, 2, 5, and 10 dpi and stored in minimum essential medium (MEM) at −80 ℃. After thawing and collecting cell suspension from NPS samples, bacteria was pelleted by centrifugation for 10 min at 3500 x g. Bacterial genomic DNA (gDNA) was isolated according to manufacturer’s instruction (QIAGEN LLC. QIAamp DNA Mini Kit 51306). Quantitative real-time PCR was performed using a ThermoFisher Scientific QuantStudio 5 Real-Time PCR machine. Primers for detecting *M. haemolytica* D153 were designed using IDT PrimerQuest™ Tool and validated using bovine NPS samples as follows: forward, 5’- TGACGGCAATCCTACAACTATC − 3’; reverse, 5’- CTACTTCCACTCCAGCTTTACC − 3’; accession number, CP005972.1. For amplification, 5 µL of gDNA was mixed with each primer, RNase-free water, and Power SYBR Green PCR Master Mix (Applied Biosystems, Carlsbad, CA).

### Ex vivo Cell Stimulation

For Toll-like receptor (TLR) agonist (innate) stimulation, we prepared complete Roswell Park Memorial Institute medium (cRPMI) following the established procedure as described in [23] with some modifications made to the experimental protocol. Fresh whole blood (200 µL/well, 4 wells) or fresh BAL cells (1 × 10^6^ cells/mL, 200 µL/well, 4 wells) were added to round-bottom, 96-well plates. The cells were stimulated with cRPMI (negative control), 1 µg/mL lipopolysaccharide (LPS) B4, 10 µg/mL Pam3CSK4, or a mixture of 50 µg/mL Poly(I: C) high molecular weight (HMW) with 10 µg/mL imiquimod. Cells were incubated for 48 h at 37 °C, 5% CO_2_. Cell culture supernatants were pooled and stored at − 80 °C.

### Flow Cytometry and Reactive Oxygen Species Assay

Fresh whole blood (200 µL/well) or fresh BAL cells (2 × 10^6^ cells/mL, 200 µL/well) were plated into a 96-well round bottom plate and centrifuged to remove the supernatant at 2000 x g for 5 min. Contaminating red blood cells were lysed by hypotonic lysis, then cells were washed, followed by surface staining as in [23]. The cells were stained with primary monoclonal antibodies (Table 2) in fluorescence-activated cell sorting (FACS) buffer (PBS with 10% FBS and 0.02% NaN_3_) and incubated for 30 min at 4 °C in the dark. After washing twice with FACS buffer, the cells were stained with secondary monoclonal antibodies and with live/dead fixable aqua dead cell stain (Table 2) for 30 min at 4 °C in the dark and then washed twice. After surface staining with fluorochrome-conjugated monoclonal antibodies (Table 2) for 30 min at 4 °C in the dark, cells were washed and then fixed with BD FACS lysis buffer (BD Biosciences, Mountain View, CA) for 10 min at room temperature. Lastly, cells were washed twice and resuspended in FACS buffer until analysis.

**Table 2:** Antibodies used for flow cytometric analysis of whole blood and BAL cells

Specificity	Target species (species cross-reactivity)	Clone	Isotype	Fluorochromes	Source	Secondary antibody, Source
Panel 1						
CD14	Bovine	CAM36A	IgG_1_	N/A	Kingfisher	PE, BioLegend
CD16	Human (Bovine)	KD1	IgG_2a_	N/A	Bio-Rad	PerCP-Cy5.5, BioLegend
CD172a	Bovine	CC149	IgG_2b_	N/A	Bio-Rad	APC-Cy7, SouthernBiotech
Granulocyte	Bovine	CH138A	IgM	N/A	Kingfisher	BV421, BioLegend
Macrophages (Calprotectin)	Human (Bovine)	MAC387	IgG_1_	APC	Bio-Rad	N/A
Panel 2						
CD11b	Bovine	MM12A	IgG_1_	N/A	Kingfisher	PerCP-Cy5.5, BioLegend
CD62L	Bovine	CC32	IgG_1_	FITC	Bio-Rad	N/A
CD172a	Bovine	CC149	IgG_2b_	N/A	Bio-Rad	APC-Cy7, SouthernBiotech
Granulocyte	Bovine	CH138A	IgM	N/A	Kingfisher	BV421, BioLegend
Panel 3						
WC 1.1 (WC1-N28 epitope)	Bovine	BAG25A	IgM	N/A	Kingfisher	PE, Invitrogen
WC 1.2 (WC1-N3 epitope)	Bovine	CACTB32A	IgG_1_	N/A	Kingfisher	AF488, SouthernBiotech
γδ TCR (γδ TCR1-N24 δ chain)	Bovine	GB21A	IgG_2b_	N/A	Kingfisher	AF647, Invitrogen
Panel 4						
CD21	Bovine	CC21	IgG_1_	FITC	Bio-Rad	N/A
CD335	Bovine	AKS1	IgG_1_	PE	Bio-Rad	N/A
CD4	Bovine	ILA11A	IgG_2a_	N/A	Kingfisher	PerCP-Cy5.5, BioLegend
CD8	Bovine	CC63	IgG_2a_	AF647	Bio-Rad	N/A

*CD*, cluster of differentiation

*AF*, Alexa Fluor

*APC*, Allophycocyanin

*Cy*, Cyanine

*FITC*, Fluorescein isothiocyanate

*PE*, Phycoerythrin

LIVE/DEAD™ Fixable Aqua Dead Cell Stain Kit to stain dead cells was used in panel 1

Zymosan and CellROX™ Green Reagent were used in panel 1

*N/A*, not applicable

For reactive oxygen species (ROS) assays, cells were stimulated with Zymosan (TLR2 agonist, 100 µg/mL) and incubated for 30 min in 37 °C incubator. Cells were then resuspended with CellROX green reagent (ThermoFisher Scientific, 2x concentration in 100 µL in cRPMI) and incubated for 30 min in the dark at room temperature. Cells were then washed and fixed with BD Cytofix fixation buffer for 30 min at 4 °C in the dark. For intracellular staining for macrophages (Calprotectin), cells were stained with anti-calprotectin antibodies for 30 min at room temperature in the dark and then washed twice with BD Perm/Wash buffer and resuspended in FACS buffer until analysis. Samples were acquired using a BD FACS Canto flow cytometer (BD Biosciences). Data were analyzed using Flowjo software version v10.8.0.

### Enzyme-Linked Immunosorbent Assay

Sandwich enzyme-linked immunosorbent assay (ELISA) was used to quantify bovine cytokines in cell culture supernatants. Cell culture supernatants were stored at −80 °C until enzyme-linked immunosorbent assay (ELISA) analysis. The concentration of cytokines in cell culture supernatants was determined using bovine commercial ELISA kits for TNFα (R&D), IL-6 (Invitrogen), and IL-1β (Invitrogen) and performed according to manufacturer’s instructions. IL-10 was measured using individual products from Bio-Rad, and the protocol and the capture and detection antibody concentrations were based on the paper [24] with modifications: 6.25 µg/mL of capture antibody (Bio-Rad), 2 µg/mL of detection antibody (Bio-Rad), and recombinant IL-10 (Bio-Rad), which ranged from 125 to 8000 pg/mL were used to detect IL-10. For detecting acute phase proteins, serum samples were separated from the whole blood. Bovine haptoglobin (Immunology Consultants Laboratory, Inc. E-10HPT) and cow C-reactive protein (Life Diagnostics, Inc. CRP-11) concentrations were determined in serum using commercial kits per manufacturer’s instruction. All samples and standards were plated in duplicates.

### Quantitative Real-time PCR

BAL cell pellets from each cattle were collected and stored in TRIzol Reagent (Invitrogen). Total RNA was extracted with chloroform and precipitated with 70% ethanol and then placed in RNeasy spin columns (QIAGEN LLC) for RNA clean-up according to the manufacturer’s instructions. The RNA concentration in each sample was determined using a Qubit RNA Broad Range Kit (Invitrogen) and measured by Qubit 4 Fluorometer (Invitrogen). RNA (500 ng per sample), random primers, and Superscript III Reverse Transcriptase were used to synthesize cDNA. Power SYBR Green PCR Master Mix (Applied Biosystems, Carlsbad, CA) was used to perform qPCR as previously described [25] with minor modifications. ThermoFisher Scientific QuantStudio 5 Real-Time PCR machine was used to run qPCR. Primers are compiled (Table 3). Where no reference is indicated, primers were designed using IDT PrimerQuest™ Tool and validated using bovine BAL cells. Primers for MMP9, MT1A, MT2A, ZIP8, IL-8, Occludin, PTGS2, and SerpinB2 were purchased from IDT. RPS9 was used as the housekeeping gene. Gene of interest copy numbers was normalized to the RPS9 housekeeping gene to correct for differences in the input material as delta cycle threshold (∆Ct).

**Table 3:** Forward and reverse primers used for qPCR

Gene	Accession #	Strand	Sequence (5’-3’)	Reference^8^
IL-8^1^	NM_173925.2	Forward	TGTGAAGCTGCAGTTCTGTCAAG	[68]
		Reverse	TGCACCCACTTTTCCTTGGGGT	
SerpinB2^2^	NM_001192051.2	Forward	CTTGGAGTTGCTGGAGAG	[69]
		Reverse	ACACTTCAGACAGAAACAGG	
MMP9^3^	NM_174744.2	Forward	GACCAGGACAAGCTCTACGG	[70]
		Reverse	CAGAAGCCCCACTTCTTGTC	
Occludin	NM_001082433.2	Forward	CTGCTGCCGACGAGTACAATAG	[71]
		Reverse	TTCCGTCGGTCGTAATCTCC	
ZIP8	NM_001205630.1	Forward	GGAGTGGAGGGAAGAAAGAAG	
		Reverse	CTCACCTCGCCTGTGTATTT	
MT-1A^4^	NM_001040492.2	Forward	ATGGACCCGAACTGCTCCTGC	
		Reverse	GCGCAGCAGCTGCACTTGTCCG	
MT-2A^5^	NM_001075140.1	Forward	ATCCTTTGCTCAGCAGTCTC	
		Reverse	ACAAACGGGTCAGGTTGTATTA	
PTGS2^6^	NM_174445.2	Forward	TCCGCCAACTTATAATGTGCAC	[72]
		Reverse	GGCAGTCATCAGGCACAGGA	
RPS9^7^	NM_001101152.2	Forward	CGCCTCGACCAAGAGCTGAAG	[73]
		Reverse	CCTCCAGACCTCACGTTTGTTCC	

^1^Interleukin-8

^2^Plasminogen activator inhibitor-2

^3^Matrix metallopeptidase 9

^4^Metallothionein 1 A

^5^Metallothionein 2 A

^6^Prostaglandin-Endoperoxide Synthase 2

^7^Ribosomal protein S9

^8^Primers were designed using IDT PrimerQuest™ Tool and validated using bovine BAL cells

### Statistical Analysis

Graphing and statistical analyses were performed using Prism v9.2.0 (GraphPad Software, Inc). Outliers were detected using the ROUT method (Prism v9.2.0) and removed from analysis. The treatment means ± the standard error of the means (SEM) was plotted, and significant differences between treatment groups were determined using a two-way ANOVA (mixed-effects model with the Geisser-Greenhouse correction) with Turkey’s multiple comparisons test. For plasma and liver TM concentration (Tables 4 and 5), data were analyzed using the mixed procedure of SAS 9.4 (Cary, NC) with the fixed effect of treatment and steer was the experimental unit. Initial plasma and liver TM concentrations were used as a covariate in subsequent analysis. For analyzing data from *M. haemolytica* detection in NPS (Table 6), ordinary one-way ANOVA with Turkey’s multiple comparisons test was used, and ordinary two-way ANOVA with Sidak’s multiple comparisons test was used for Fig. 4. For *M. haemolytica* detection in BAL (Table 6), the GLIMMIX procedure of SAS 9.4 (Cary, NC) was used. The data were analyzed as binary data, and the model included the fixed effect of treatment. Statistical significance was determined at *P* ≤ 0.05, and a statistical tendency was determined at 0.05 < *P* ≤ 0.1.

**Table 4:** Plasma trace mineral (TM) concentration in steers receiving an ITM injection on day −6 (ITMPRE) or day 2 (ITMPOST) post infection with *M. haemolytica* strain D153

Plasma	Treatments			SEM^1^	Treatment
TM concentration	CON	ITMPRE	ITMPOST		*P*-value
Steers (*n*)	9	12	11		
Zn, mg/L					
Day 0	1.09	1.11	1.18	0.05	0.41
Day 2	1.01	0.95	0.99	0.06	0.78
Day 5	1.14	1.13	1.11	0.04	0.80
Cu, mg/L					
Day 0	1.02	1.03	1.07	0.06	0.82
Day 2	1.07	1.04	1.00	0.04	0.50
Day 5	1.18	1.13	1.04	0.06	0.25
Se, ug/L					
Day 0	124	130	124	7.8	0.78
Day 2	113	116	109	5.6	0.57
Day 5	124	118	132	6.6	0.26
Mn, ug/L					
Day 0	1.88	1.96	1.77	0.11	0.38
Day 2	1.81	1.66	1.70	0.09	0.44
Day 5	1.87^b^	1.69^c^	2.03^a^	0.07	0.001

^1^Highest SEM of any treatment

^a, b,c^Within a row, treatment means with different superscripts differ *P ≤* 0.05

**Table 5:** Liver trace mineral (TM) concentration in steers receiving an ITM injection on day −6 (ITMPRE) or day 2 (ITMPOST) post infection with *M. haemolytica* strain D153

Liver	Treatments			SEM	Treatment
TM concentration, mg, kg DM	CON	ITMPRE	ITMPOST		*P*-value
Steers (*n*)	9	12	11		
Zn					
Day −10	104	106	116	8.55	0.54
Day 2	109	117	115	8.43	0.72
Day 10	100	112	102	8.37	0.42
Cu					
Day −10	203	201	242	25.42	0.37
Day 2	190^b^	251^a^	198^b^	12.71	0.001
Day 10	194	248	215	21.15	0.14
Se					
Day −10	1.79	1.69	1.86	0.10	0.45
Day 2	1.48^b^	3.05^a^	1.67^b^	0.24	0.0001
Day 10	1.72^b^	2.65^a^	2.33^ab^	0.17	0.001
Mn					
Day −10	9.79	9.03	9.27	0.66	0.68
Day 2	8.04	8.37	7.98	0.40	0.67
Day 10	9.06	9.28	7.52	0.94	0.25

^1^Highest SEM of any treatment

^a, b^Within a row, treatment means with different superscripts differ *P ≤* 0.05

**Table 6:** *M. haemolytica* positive detection in cattle from qualitative culture of BAL samples and qPCR on nasopharyngeal swab samples

* M. haemolytica* detection	Treatments			SEM^1^	*P*-value
	CON	ITMPRE	ITMPOST		
Steers (*n*)	9	12	11		
BAL^2^					
Day 0	4/9 (44.4%)	3/12 (25.0%)	3/11 (27.3%)	16.6	0.61
Day 2	6/9 (66.7%)	3/12 (25.0%)	3/11 (27.3%)	15.7	0.14
Day 5	1/9 (11.1%)	3/12 (25.0%)	2/11 (18.2%)	12.5	0.73
Day 10	2/9 (22.2%)	4/12 (33.3%)	3/11 (27.3%)	13.9	0.85
NPS^3^					
Day 0					
Ct mean^4^	31.48	30.46	29.22	1.62	0.61
Positive sample #	7/9 (77.8%)	9/12 (75.0%)	8/11 (72.7%)		
Day 2					
Ct mean^4^	31.63	29.23	29.97	1.84	0.69
Positive sample #	3/9 (33.3%)	8/12 (66.7%)	8/11 (72.7%)		
Day 5					
Ct mean^4^	34.96	34.90	35.34	0.71	0.79
Positive sample #	4/9 (44.4%)	6/12 (50.0%)	5/11 (45.5%)		

^1^Highest SEM of any treatment

^2^Qualitative culture by plating BAL samples

^3^qPCR on nasopharyngeal swab samples

^4^Average of Ct values from *M. haemolytica* positive samples

## Results and Discussion

### Plasma and Liver Trace Mineral Concentrations in Steers Receiving an ITM Injection at Processing or During Antibiotic Treatment Following *M. haemolytica* Infection

Plasma TM concentrations were analyzed at 0, 2, and 5 dpi (Table 4). Notably, 0 dpi corresponds to 6 days post-ITM treatment for ITMPRE and 2 days prior to treatment for ITMPOST. The steers were maintained on a forage-based diet for the duration of the study that was not supplemented with Mn, Zn, Cu, or Se. As seen in Table 1, the diet met NRC requirements for Mn, Zn, and Se (defined as 20, 30, and 0.10 mg/kg DM, respectively [26]) but was deficient for Cu (defined as 10 mg/k DM) [26]. There were no treatment effects on plasma Zn, Cu, or Se, but we observed ITMPOST had increased MN compared to ITMPRE and CON at 5 dpi (*P ≤* 0.05). Our own previous research, as well as others, have shown that in healthy steers, plasma concentrations of Zn and Mn peak rapidly after ITM injection, but return to concentrations similar to untreated controls by 24–48 h post-treatment [27, 28]. Se also peaks at 8–10 h after ITM injection but remains elevated longer, returning to baseline by day 8 [27, 28]. TM concentrations are rapidly decreased in plasma because they are redirected to liver for storage [27] or other locations in the body where they are needed [28]. In contrast, one study in cross-bred beef steers found that ITM injection concurrent with a multivalent, modified-live vaccine resulted in an increase in serum Zn, Cu, and Se for as long as 14 days after administration [5]. The reason for the prolonged impact on circulating trace mineral concentrations in this study is unclear but may be due to the effect of concurrent vaccination or the trace mineral status of the animals at the time of enrollment. The effects of inflammation on plasma trace mineral concentrations, particularly Zn, are well described [15]. Inflammation induces sequestration of Zn and Se from circulation to reduce access to invading pathogens. In one study, Holstein calves infected with *M. haemolytica* had significantly reduced plasma Zn concentrations at 1, 2, and 3 dpi compared to the uninfected controls group [29]. Our findings are consistent with these previous studies and align with a nutritional immunity response, as all groups of animals experienced decreases in plasma Zn, Se, and Mn at 2 dpi.

Liver TM concentrations were determined on days −10, 2, and 10 relative to *M. haemolytica* infection (Table 5). Day −10 was prior to ITM treatment for all cattle, while 2 dpi is 8 days after ITM treatment for ITMPRE and immediately prior to ITM treatment for ITMPOST. Cu concentration was higher in ITMPRE (*P ≤* 0.05) than ITMPOST and CON at 2 dpi. Se concentration was also higher in ITMPRE (*P ≤* 0.05) compared to ITMPOST and CON at 2 dpi and remained elevated at 10 dpi compared to CON. Liver Se did not differ between ITMPRE and ITMPOST at 10 dpi, but their concentrations were higher than CON (*P ≤* 0.05) at 10 dpi. No treatment differences were detected in liver Zn (*P >* 0.10) or Mn (*P >* 0.10) concentration. In our previous work, ITM injection improved liver Cu and Se concentrations in feedlot steers for at least 29 days compared to saline-treated controls [28]. Similarly, [27] observed improved liver Cu and Se in ITM-injected animals for at least 15 days, while [30] observed increased Se concentrations in liver for 15 days but no effects on liver Cu. Palomares et al. observed that administration of ITM increased liver Se (on days 21 and 56), Cu (on day 56), and Mn (on day 56) compared to saline injections [6]. In the current study, ITM injection maintained stable liver Se concentrations in both ITMPRE and ITMPOST animals, but did not impact liver Cu, Zn, or Mn. In contrast, *M. haemolytica* challenge appeared to impact both Se and Mn liver concentrations, causing a decrease from day −10 to 2 dpi. However, this must be interpreted with caution as the timeframe between the pre-challenge liver biopsy and the experimental infection was quite long and included a transport stress event. Without an uninfected control group, it is difficult to know if the changes in Se and Mn are attributable to the challenge alone or additional factors. Little is known regarding liver Mn status during acute inflammation, although one study using mouse models of both acute intestinal inflammation and systemic *Candida albicans* infection observed a rapid decline in liver Mn concentration [31]. It seems likely that Mn and Se may be transported from the liver to other parts of the body to support the ongoing immune response. Alternatively, as a powerful antioxidant [32], Se may play a role in preventing inflammation-associated oxidative stress.

### Reduced Clinical and TUS Scores in Steers Receiving an ITM Injection At Processing or During Antibiotic Treatment Following *M. haemolytica* Infection

**Fig. 2:**
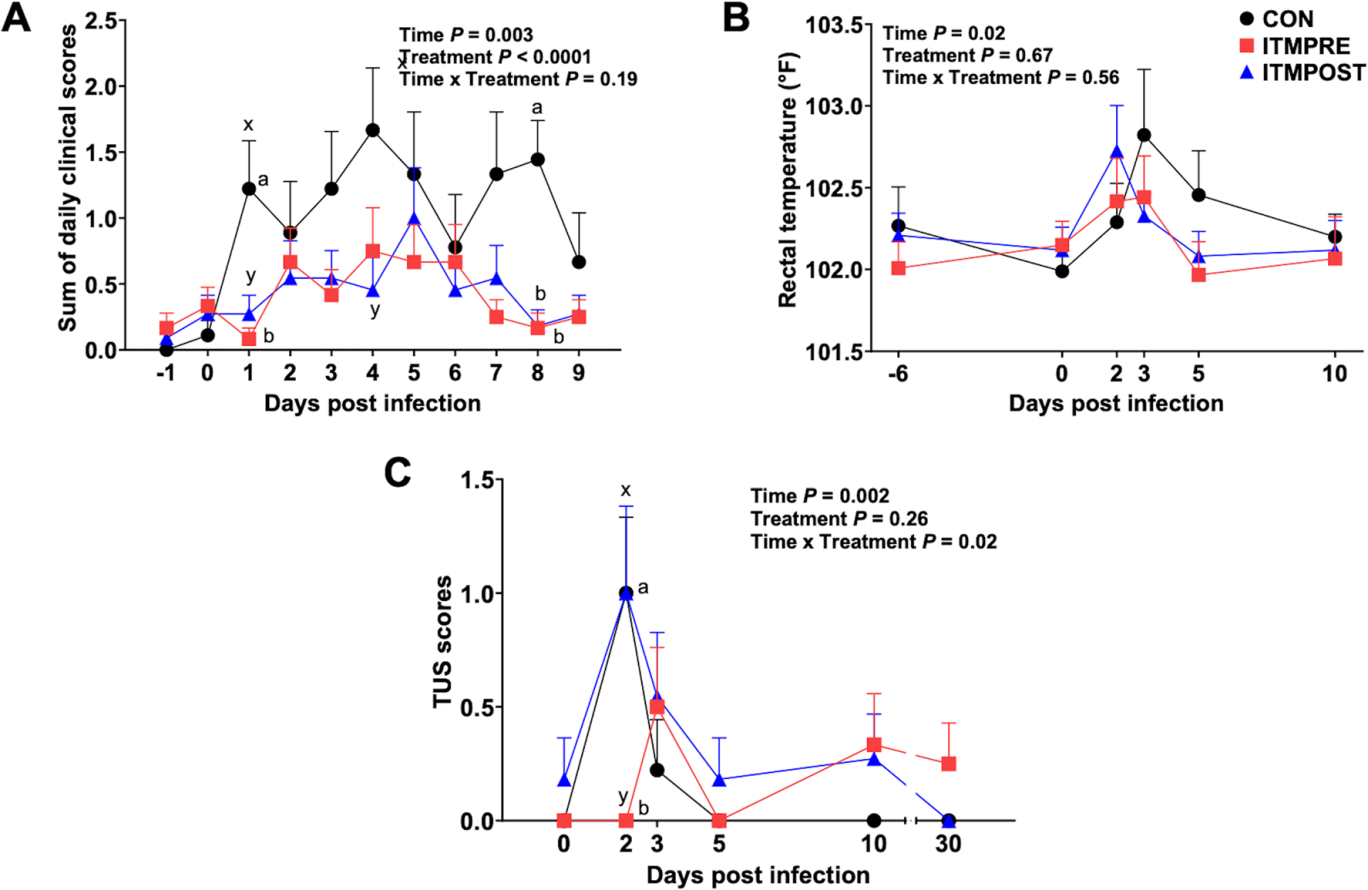
Effects of ITM treatment on clinical disease, rectal temperature, and thoracic ultrasonography (TUS) scores following a *M. haemolytica* challenge. ITMPRE was administrated with ITM 6 days before *M. haemolytica* infection. ITMPOST was injected with ITM concurrently with an antibiotic at 2 dpi of *M. haemolytica*. CON received sterile saline as control. **A** Clinical examination was performed once daily from − 1 through 9 dpi by a trained observer using the DART scoring system. **B** Rectal temperature was measured once on −6, 0, 2, 3, 5, and 10 dpi. **C** Thoracic ultrasonography (TUS) was performed on 0, 2, 3, 5, 10, and 30 dpi with the *M. haemolytica*-challenged cattle to measure and monitor the degree of lesions and consolidation in the lung. Data represent means ± SEM. Data were analyzed using a two-way ANOVA (mixed-effects model with the Geisser-Greenhouse correction) with Turkey’s multiple comparisons test. ^a, b^The significant difference between groups on each dpi, treatment means with different superscripts differ *P* < 0.05. ^x, y^Tendency between groups on each dpi, treatment means with different superscripts differ 0.05 < *P* < 0.1

Clinical scores were evaluated daily by a trained observer that was blinded to treatment groups (Fig. 2A). Clinical scores included coughing and nasal discharge in most animals, with some developing reduced attitude scores. Clinical disease scores changed over time for all groups following *M. haemolytica* challenge (time effect, *P* < 0.01) and were less pronounced in the ITM-treated groups (*P* < 0.0001) compared to CON. ITMPRE had lower clinical scores compared to CON (*P* < 0.05) at 1 and 8 dpi. ITMPOST had lower clinical scores on days 1, 4 (*P* < 0.1), and 8 dpi (*P* < 0.05) compared to CON. No treatment differences were noted for rectal temperatures (Fig. 2B), although all groups developed elevated temperatures at 2 to 3 dpi that persisted in some animals to 5 dpi. Longitudinal TUS was used to evaluate the degree of lung consolidation following *M. haemolytica* infection. Overall, the amount of lung damage observed by TUS was mild and included mostly “comet tails,” small perturbances in the ultrasound likely representative of pulmonary edema, with a few animals containing small areas of consolidation [17]. As seen in Fig. 2C, there was a time × treatment interaction with ITMPRE developing lower TUS scores compared to CON (*P* < 0.05) and ITMPOST (*P* < 0.1) at 2 dpi. Two animals in ITMPRE developed a TUS score of 2 at 10 dpi, but lesions were not present on day 5 dpi (Fig. 2C). Due to this delay in lung lesion kinetics relative to other animals, it is not clear if the lesions developed due to the *M. haemolytica* infection or possibly a secondary infection.

Administering ITM to stressed calves upon arrival has been shown to improve their health during the crucial initial receiving phase. One field study comparing two ITM products containing Cu, Se, Mn, and Zn showed that beef heifers receiving either ITM on day 1 after arrival had lower BRD morbidity rates compared to untreated controls [33]. Animals receiving ITM1 and ITM2 had a 54.8% and 67.9% morbidity rate, respectively, while the controls had a morbidity rate of 87.1% [33]. In another study, the sum of daily health scores was examined in newly received beef calves challenged with BVDV2 5 days after receiving ITM treatment concurrently with an MLV vaccination [13]. Calves receiving ITM treatment concurrent with vaccination had improved health scores compared to unvaccinated control calves, although they were not significantly different from vaccinated without ITM group from 7 to 9 dpi of BVDV2 [13]. Our results are consistent with both blocks, in that we observed a positive impact of ITM treatment on clinical disease scores in calves experimentally infected with *M. haemolytica* (Fig. 2A). In the current study, clinical disease scores were consistently lower in the ITM-treated cattle compared to CON throughout infection, although rectal temperatures did not differ between treatments. The DART system is a 4-point scoring system that evaluates depression, appetite, respiration, and temperature. If cattle present with a DART score of 2 and a rectal temperature of 40 °C, or a DART score of 3, regardless of temperature, they are recommended for BRD treatment [16, 34]. In the current study, we chose to treat all animals with an antibiotic at 2 dpi, regardless of clinical disease score. However, if we instead applied the DART criterion to make treatment decisions, a total of 12 steers would have required antibiotic treatment during the study. Of those, 7 head were CON, 3 were ITMPOST, and 2 were ITMPRE.

Based on clinical disease parameters alone, it is difficult to conclude if ITM administration at processing is more effective than administration at the time of BRD treatment. However, by also considering the TUS scores, our results support the use of ITM in steers at the time of receiving processing. Longitudinal TUS is a noninvasive approach to detect and score lung lesions and is often used on-farm for detection of subclinical BRD due to its increased sensitivity over other diagnostic approaches [17, 35]. The TUS scoring system we used has a maximum 4-point scoring system. Comet tails in the absence of lung consolidation results in a score of 1. Lung consolidation, a more serious lesion, results in a score of at least 2, with increasing scores based on the amount of visual field affected [17]. In the current study, ITMPRE maintained lower TUS scores compared to either CON or ITMPOST (ITM treatment at 2 dpi). No ITMPRE animals received a TUS score greater than 2, while multiple animals in CON and ITMPOST received scores of up to 3. It is important to note that the experimental infection in the current study was relatively mild and caused no mortality. Thus, the benefit of ITM may be even more pronounced if used in animals experiencing a more severe BRD challenge or in animals that were deficient in one or more trace minerals. Together, our results suggest that calves experiencing BRD may benefit from ITM treatment regardless of when it is administered, but ITM injection at processing may position the animal to be more resilient to a subsequent BRD event.

### *Mannheimia haemolytica* Detection in BAL and NPS from Steers Receiving an ITM Injection at Processing or During Antibiotic Treatment Following *M. haemolytica* Infection

To monitor bacterial burden, we conducted qualitative culture for *M haemolytica* on BAL samples at 0, 2, 5, and 10 dpi and qPCR on NPS at 0, 2, and 5 dpi. No treatment effects were observed for either sample type (Table 6).

*M. haemolytica* is commonly identified in the upper respiratory tract and nasopharynx of healthy cattle as a commensal organism; thus, the observation that animals were positive for *M. haemolytica* in the NPS at 0 dpi was not unexpected [36]. However, stress or infection with other microorganisms can lead to overgrowth of *M. haemolytica*, leading to invasion of the lower respiratory tract and development of pneumonia [36]. In the current study, cattle were experimentally infected by intratracheal inoculation. Thus, the bacteria were instilled below the NPS to encourage establishment in the lung, and it may not be surprising that we did not observe differences in NPS recovery between groups. Others have shown that quantitative culture from BAL does not always correlate with bacterial pneumonia in calves. Some clinical calves may be negative for bacterial recovery, while healthy calves may be positive by culturing due to normal colonization in the respiratory tract or possible contamination by passing the tube through the nasopharynx [37]. Poor recovery from infected animals may also be due to challenges washing the bacteria out of airways that are already inflamed or occluded due to the infection. Additionally, in the current study, we encountered some technical challenges with detection of *M. haemolytica* in BAL. For logistical reasons, BAL was suspended in sucrose and frozen for later culturing, rather than freshly cultured. This likely resulted in underestimation of bacterial load in the lungs. Despite these challenges, while not statistically significant, it is interesting to note that *M. haemolytica* was recovered from the lungs of more than half of CON calves, but only 25–30% of the animals in the ITM-treated groups.

### Induction of Acute Phase Response Proteins Following *M. haemolytica* Infection

**Fig. 3:**
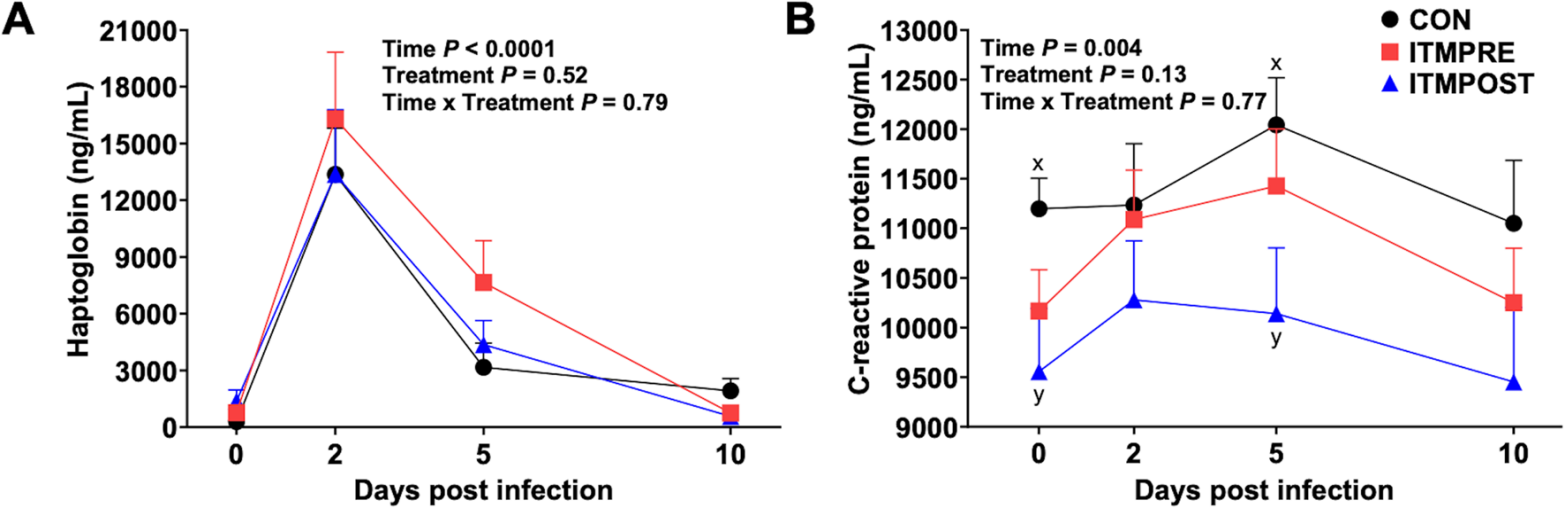
Concentrations of **A** haptoglobin (Hp), **B** C-reactive protein (CRP) in serum of cattle before and after application of ITM or antibiotic during *M. haemolytica* infection. Blood was collected via the jugular vein on 0, 2, 5, and 10 dpi, and serum was separated and collected for measuring haptoglobin and CRP concentration using commercial ELISA kits. Graph represents means ± SEM. Data were analyzed using a two-way ANOVA (mixed-effects model with the Geisser-Greenhouse correction) with Turkey’s multiple comparisons test. ^x, y^Tendency between groups on each dpi, treatment means with different superscripts differ 0.05 < *P* < 0.1

Hp is a major acute phase protein and a sensitive marker for assessing and monitoring respiratory disease in feedlot cattle [38]. We observed a time effect (*P* < 0.0001), with Hp peaking sharply at 2 dpi and declining by 5 and 10 dpi (Fig. 3A), indicating an increase in Hp due to bacterial infection and subsequent decline after antibiotic treatment at 2 dpi. However, no treatment effects (*P* = 0.52) were observed for Hp concentration. Our results are consistent with Caramalac et al. (2021), who observed no differences in Hp concentration between ITM and saline treated feedlot heifers challenging with porcine red blood cells concurrent with ITM administration [30]. CRP is a moderate acute phase protein in cattle [38] that is also elevated in cattle experiencing BRD [39]. In the current study, CRP followed a slightly different pattern than Hp, with a gradual increase from 2 to 5 dpi and decline by 10 dpi (time effect, *P* = 0.004). The concentration of CRP tended to be reduced in ITMPOST on 5 dpi compared to CON, but no additional treatment effects were observed (*P* = 0.13). This may indicate some differences in the overall magnitude of the inflammatory response in ITM-infected cattle, although CRP concentrations appear to be less sensitive in predicting clinical disease severity in cattle compared to Hp [38].

### Flow Cytometry Analysis of Immune Cell Subsets in Blood and BAL from Steers Receiving an ITM Injection at Processing or During Antibiotic Treatment Following *M. haemolytica *Infection

The frequencies of circulating myeloid cell subsets were evaluated by flow cytometry on whole blood on 0, 2, and 5 dpi (Table 7). The frequency of total CD172^+^ monocytes and neutrophils increased from 0 to 5 dpi and peaked at 5 dpi (*P* < 0.0001), although no treatment effects were observed (*P* > 0.1). The frequencies of lymphocyte subsets were also evaluated on 0, 2, and 5 dpi, but we detected no time or treatment effects in the frequencies of circulating lymphocyte populations (*P >* 0.10). The rapid increase in both neutrophils and monocytes is consistent with other experimental infection models of *M. haemolytica* [40, 41]. Bacterial infection induces activation of the acute phase response and systemic inflammatory responses, which results in increased release of innate immune cells from the bone marrow to the blood to fight the infection.

**Table 7:** Effects of ITM treatment on the frequency of myeloid cells in blood of steers experiencing a *M. haemolytica* infection

Whole blood	Treatments			SEM^1^	Time	Treatment	Time × Treatment
Freq. of live	CON	ITMPRE	ITMPOST		*P*-value	*P*-value	*P*-value
Steers (*n*)	9	12	11				
Monocytes, %							
Day 0	0.70	1.66	1.54	0.40	< 0.0001	0.29	0.58
Day 2	2.25	1.90	2.67	0.33			
Day 5	3.43	3.70	4.18	0.64			
cM^2^, %							
Day 0	0.62	1.48	1.31	0.37	< 0.0001	0.28	0.60
Day 2	2.08	1.78	2.50	0.31			
Day 5	3.02	3.36	3.80	0.60			
intM^3^, %							
Day 0	0.012	0.069	0.131	0.056	0.03	0.098	0.11
Day 2	0.018	0.015	0.023	0.003			
Day 5	0.019	0.017	0.023	0.006			
ncM^4^, %							
Day 0	0.036	0.045	0.061	0.016	< 0.0001	0.066	0.045
Day 2	0.085	0.050	0.071	0.019			
Day 5	0.205^x^	0.107^y^	0.167	0.034			
Neutrophils^5^, %							
Day 0	9.06	12.41	8.39	3.50	< 0.0001	0.52	0.65
Day 2	17.77	22.84	16.27	4.32			
Day 5	19.95	24.02	22.86	3.01			

^1^Highest SEM of any treatment

^2^*cM*, classical monocytes

^3^*intM*, intermediate monocytes

^4^*ncM*, nonclassical monocytes

^5^*PMN*, polymorphonuclear leukocyte

^x, y^Within a row, treatment means with different superscripts differ 0.05 < *P* < 0.1

We further analyzed monocyte subsets based upon their expression of CD14 and CD16 [42]. Classical monocytes (cM; CD14 + CD16−) increased in frequency from 0 to 5 dpi, but there were no differences due to treatment (*P =* 0.29). However, the frequency of both intermediate (intM; CD14 + CD16+) and non-classical (ncM; CD14−CD16+) monocyte subsets tended to differ in response to ITM treatment. The intM subset decreased from 0 to 2 dpi in ITMPRE and ITMPOST compared to CON. The ncM subset of monocytes tended to be higher in CON compared to ITMPRE at 5 dpi (0.05 < *P* < 0.1). Few studies in cattle have explored the function or impact of infection on circulating monocyte subsets; however, the importance of these subsets in human and rodent models has been more widely explored [43]. Classical monocytes produce great quantities of reactive oxygen species and proinflammatory cytokines and express many chemokine receptors, making them important effector cells in tissues [44]. Nonclassical monocytes, in contrast, are anti-inflammatory and are associated with wound healing and endothelial cell homeostasis [45]. The functional role of intM monocytes is less understood, although this population expresses the highest levels of molecules needed for antigen-presentation and is expanded in the blood of patients with systemic infection [43]. In humans hospitalized with SARS-Cov2 infection, elevated frequencies of ncM cells have been shown to correlate with clinical disease severity, perhaps indicating a compensatory change in the immune system to promote resolution of inflammation or lung healing [46]. In the present study, we observed a tendency for both intM and ncM subsets to be higher in CON compared to ITM-treated groups, which may be related to the reduced clinical disease observed in the ITMPRE and ITMPOST. Because very little is known about the role of individual monocyte subsets in cattle with respiratory disease, this will be an interesting area of future research.

The frequencies of immune cells in the BAL were also evaluated by flow cytometry on 0, 2, and 5 dpi. The frequency of alveolar macrophages changed over time (*P* = 0.0001), decreasing from day 0 to 2 dpi, then increasing from 2 to 5 dpi. On the other hand, neutrophils in BAL increased from day 0 to 2 dpi, peaked at 2 dpi, and decreased from day 2 to 5 post infections (*P* = 0.0002), although no treatment effects were observed (*P* > 0.1). Around 90% of the cellular composition in BAL fluid in healthy calves comprises alveolar macrophages, with the remaining population consisting of neutrophils, lymphocytes, and epithelial cells. Abutarbush et al. (2019) used cytology to enumerate cells from the BAL of healthy adult cattle. Alveolar macrophages constituted approximately 94.3% (± 3.9) of the total cell population. Neutrophils comprised 4.3% (± 3.9), followed by lymphocytes at 1.3% (± 1.2) and rare to no frequencies of eosinophils [47]. This result is consistent with our results from flow analysis. After *M. haemolytica* infection, acute pneumonia is marked by the infiltration of the airways with an inflammatory exudate, comprising neutrophils, fibrin, and blood in the lung [48]. Following the bacterial infection, the percentage of alveolar macrophages in the airways declines proportionally due to infiltration and accumulation of neutrophils [48, 49]. This is consistent with our observations in the current study, whereby the frequency of alveolar macrophages declined from day 0 to 2 dpi, while neutrophils in BAL increased from day 0 to 2 dpi. By 5 dpi, the ratio of neutrophils and alveolar macrophages started to return to normal levels.

To determine if ITM treatment impacted cellular activation, we evaluated expression (MFI) of the activation markers CD14, CD11b, CD62L, and calprotectin on alveolar macrophages and neutrophils from BAL (Supplementary data). Expression of CD14 was reduced on neutrophils from ITMPRE compared to CON on days 0 (*P* < 0.1) and 5 dpi (*P* < 0.05) and on ITMPOST compared to CON at 5 dpi. Expression of CD11b tended to be higher (*P* < 0.1) in alveolar macrophages from ITMPRE compared to CON at 5 dpi but did not differ at any other timepoint. No treatment or time effects were observed for CD62L or calprotectin MFI. We also measured oxidative burst capacity by CellROX MFI, but also did not observe any treatment or time effects for these parameters (Supplementary data). Together, these results suggest that ITM treatment may impact recruitment of cells to the BAL but does not specifically impact their activation status or functional capacity.

Interestingly, as seen in Table 8, the frequency of both γδ T cells and NK cells in the BAL differed by treatment. The frequency of total γδ T cells was highest in ITMPRE compared to ITMPOST and CON at 5 dpi, and both ITM groups were higher than CON. This change appeared to be driven by differences in the frequency of both the WC1.2^+^ and DN subsets of γδ T cells which were elevated in both ITMPRE and ITMPOST at 5 dpi compared to CON. The frequency of NK cells was also elevated (*P* < 0.05) in both ITMPRE and ITMPOST compared to CON at 5 dpi. There is little known about the relationship between γδ T cells and trace mineral status. In cattle, γδ T cells comprise a significant population in the tissues and peripheral blood [50]. They serve as a bridge between innate and adaptive immunity and are important for barrier function. While WC1.1^+^ γδ T cells are proinflammatory and play a role in Th1-associated immunity [51], WC1.2^+^ and DN γδ T cells are regulatory in cattle [52, 53]. In the lungs of animals infected with *M. haemolytica*, the increased prevalence of WC1.2^+^ and DN subsets in ITM-treated animals may indicate an anti-inflammatory or tissue-repairing environment. Previous work in humans has shown that NK cell frequency and function is closely linked to Zn status, with Zn sufficient individuals having greater frequencies of NK cells and improved antiviral capacity [54, 55]. Similarly, Se status has also been shown to positively correlate with NK cell function and cytokine secretion [56]. Taken alone, the increase in NK cells in the BAL of cattle infected with *M. haemolytica* may suggest an enhanced capacity for proinflammatory cytokine secretion or antiviral protection. Interestingly, however, there is emerging evidence suggesting that subsets of NK cells in the lung may also serve important roles in tissue maintenance and repair [57, 58]. Coupled with the increase in regulatory γδ T cells, the increase in NK cell frequency could instead be promoting tissue repair in the lungs of ITM-treated animals.

**Table 8:** Effects of ITM treatment on the frequency of immune cells in BAL of steers experiencing a *M. haemolytica* infection

BAL	Treatments			SEM^1^	Time	Treatment	Time × Treatment
Freq. of live	CON	ITMPRE	ITMPOST		*P*-value	*P*-value	*P*-value
Steers (*n*)	9	12	11				
Alveolar macrophages, %							
Day 0	85.66^a, x^	76.15^b^	77.75^y^	2.38	0.0001	0.38	0.86
Day 2	61.53	57.68	61.74	10.7			
Day 5	83.04	70.63	76.44	4.57			
Neutrophils, %							
Day 0	0.88^y^	3.45	2.89^x^	1.29	0.0002	0.18	0.74
Day 2	9.27	18.48	13.84	4.64			
Day 5	0.96^b, y^	4.36^a^	3.00^x^	1.01			
γδ T cells, %							
Day 0	1.11	1.85	1.40	0.31	0.74	0.007	0.33
Day 2	1.23	1.51	1.17	0.33			
Day 5	0.68^b^	2.09^a^	1.37^a^	0.36			
WC 1.2 (γδ T cells), %							
Day 0	0.30	0.49	0.46	0.11	0.63	0.24	0.45
Day 2	0.53	0.53	0.44	0.22			
Day 5	0.25^b^	0.60^a^	0.58^a^	0.11			
WC 1.1 (γδ T cells), %							
Day 0	0.03	0.04	0.04	0.01	0.05	0.997	0.62
Day 2	0.04	0.04	0.05	0.01			
Day 5	0.03	0.03	0.02	0.01			
DN^2^ (γδ T cells), %							
Day 0	0.54^y^	0.98^x^	0.71	0.16	0.95	0.017	0.27
Day 2	0.62	0.89	0.65	0.15			
Day 5	0.38^b, y^	1.13^a^	0.68^x^	0.20			
B cells, %							
Day 0	1.68	2.74^a^	1.39^b^	0.46	0.96	0.26	0.27
Day 2	2.08	1.84	1.71	0.56			
Day 5	1.92	2.05	1.78	0.42			
CD4 + cells, %							
Day 0	0.79	1.12	0.59	0.29	< 0.0001	0.16	0.36
Day 2	2.44	3.58	2.77	0.90			
Day 5	0.73	3.07	0.98	1.15			
CD8 + cells, %							
Day 0	1.12	1.92	1.78	0.34	0.05	0.15	0.24
Day 2	2.48	2.55	1.72	0.93			
Day 5	1.54^b^	3.31^a^	3.02	0.69			
NK cells, %							
Day 0	0.33	0.56	0.33	0.11	0.19	0.05	0.29
Day 2	0.57	0.65	0.45	0.20			
Day 5	0.16^b^	0.63^a^	0.48^a^	0.11			

^1^Highest SEM of any treatment

^2^Double negative population

^a, b^Within a row, treatment means with different superscripts differ *P* < 0.05

^x, y^Within a row, treatment means with different superscripts differ 0.05 < *P* < 0.1

### Enhanced IL-6 Cytokine Production in the Blood of Steers Receiving ITM

**Fig. 4:**
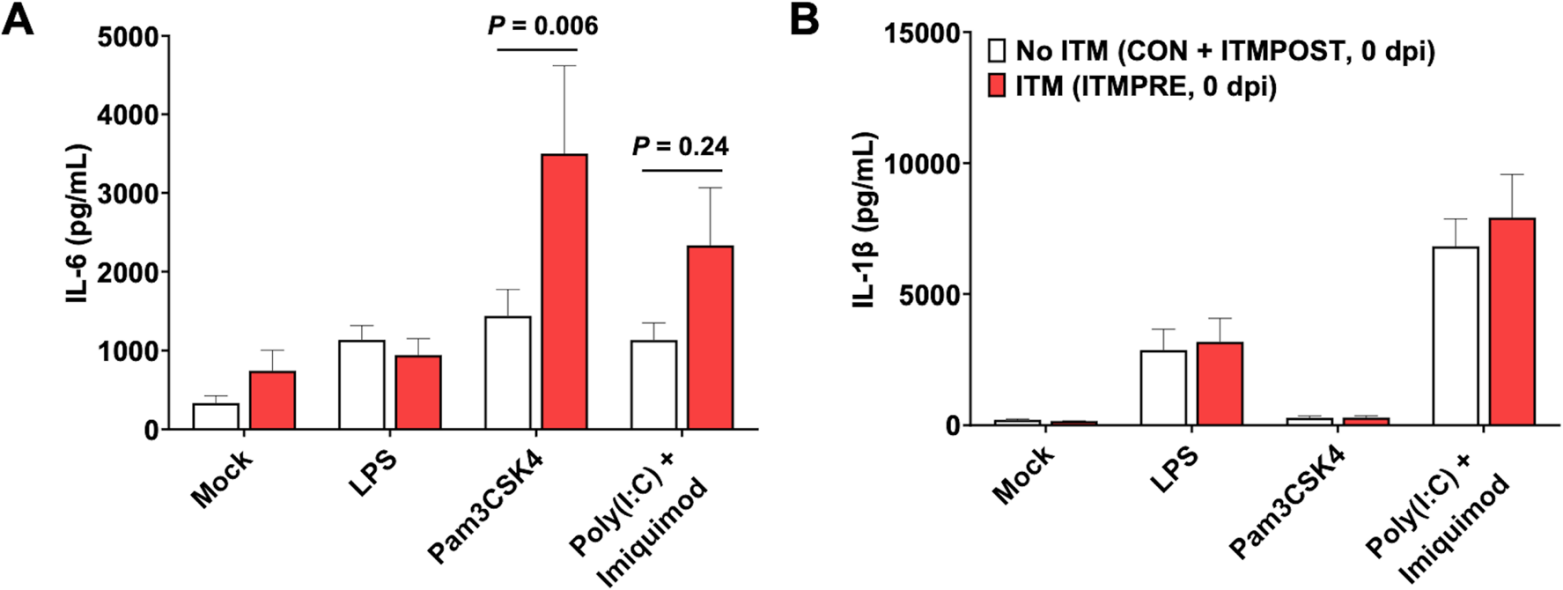
Enhanced IL-6 cytokine production by whole blood cells in steers receiving an ITM injection before bacterial infection. Whole blood was collected from steers (*n* = 32) that were treated with ITM 6 days before (ITMPRE, red bars) or untreated (comprised CON and ITMPOST) immediately before *M. haemolytica* infection (0 dpi). Cells were plated at 2 $\times$ 10^5^ cells/well in 96-well plates. Cells were stimulated for 48 h with 1 µg/mL LPS, 10 µg/mL Pam3CSK4, or a mixture of 50 µg/mL Poly(I: C) with 10 µg/mL Imiquimod. Mock wells were media only. After 48 h, cell culture supernatants were collected and analyzed by commercial ELISA kit to detect IL-6 and IL-1β. Graph represents means ± SEM. Data were analyzed using a two-way ANOVA (mixed-effects model with the Geisser-Greenhouse correction) with Turkey’s multiple comparisons test

The capacity for increase proinflammatory cytokine secretion by circulating innate immune cells has been shown to correlate with improved disease resistance in calves and other species [18, 23, 59]. Given the observed changes in clinical disease in ITM-treated steers, we measured TLR-induced proinflammatory cytokine secretion in whole blood samples collected prior to *M. haemolytica* infection (6 days post-ITM administration). Whole blood was stimulated with LPS (a TLR4 agonist), Pam3CSK4 (a TLR2/1 agonist), or Poly(I: C)/Imiquimod (TLR3 and TLR7/8 agonists, respectively). As seen in Fig. 4A, leukocytes from ITMPRE steers produced more (*P* < 0.01) IL-6 in response to Pam3CSK4 than cells from the non-ITM-treated groups (CON and ITMPOST). However, we observed no differences in the response to LPS or Poly(I: C)/Imiquimod with Imiquimod for IL-6 production at this time point or differences in the production of IL-1 β between treatment groups. In a parallel set of assays, we observed no differences in the capacity for BAL cells to generate proinflammatory cytokines (IL-1β, IL-6, IL-10, TNFα) prior to experimental infection. In previous work, we have observed that circulating cells from preweaned calves receiving a postbiotic supplement have an improved capacity to produce proinflammatory cytokines, and this correlates with improved resistance to a subsequent viral or viral/bacterial coinfection [18, 23, 59]. Thus, the increased capacity for IL-6 secretion from ITM-treated animals may be one factor promoting immune readiness in calves exposed to a respiratory infection. One recent study in humans showed that low levels of serum Zn, even if these values were considered clinical sufficient, correlated with a reduced cytokine responses from whole blood samples stimulated with TLR2/1 ligands [60]. This suggests that, even if an animal is considered nutritionally sufficient for certain trace minerals, this may not be sufficient to mount an optimum innate immune response. In these instances, ITM injection may provide additional support.

### BAL Cell Gene Expression

**Fig. 5:**
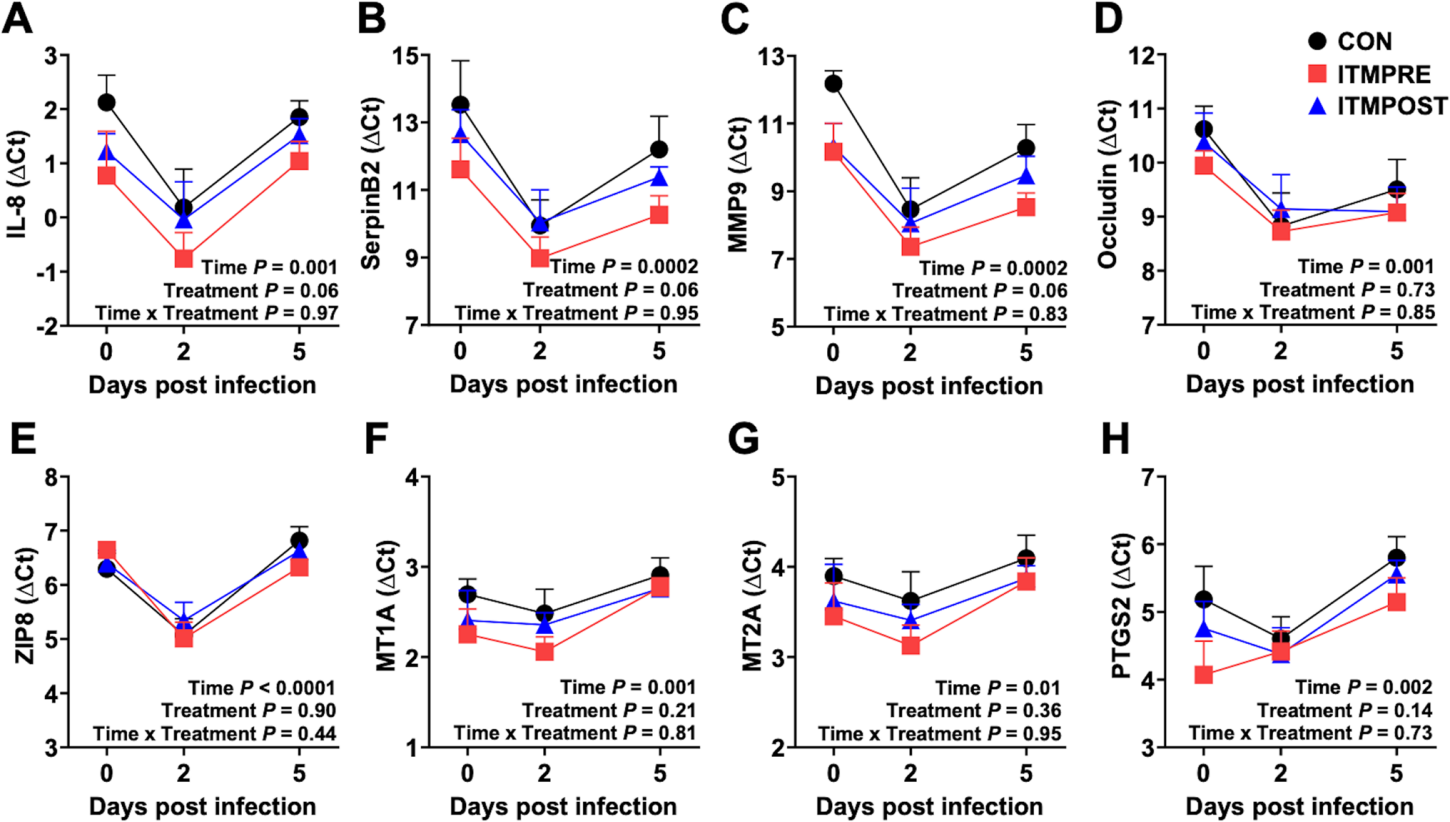
Gene expression by BAL cells from steers responding to *M. haemolytica* infection and ITM treatment. Steers (*n* = 32) were infected with *M. haemolytica*, followed by day 2 antibiotic treatment. ITM was administered either 6 days before infection (ITMPRE, red squares) or 2 dpi (ITMPOST, blue triangles). Controls (CON, black circles) were untreated with ITM. BAL cells were collected from all treatment groups at 0, 2, and 5 dpi and analyzed by qPCR for interleukin-8 (IL-8), plasminogen activator inhibitor-2 (SerpinB2), matrix metallopeptidase 9 (MMP9), occludin, ZIP8, metallothionein 1 A (MT1A), metallothionein 2 A (MT2A), and prostaglandin-Endoperoxide Synthase 2 (PTGS2). The results were normalized to the housekeeping gene RPS9 and are shown as ∆Ct. Lower ∆Ct means a higher gene expression level. Data represent means ± SEM and were analyzed using a two-way ANOVA (mixed-effects model with the Geisser-Greenhouse correction) with Turkey’s multiple comparisons test

To further explore effects of ITM treatment on the immune response in the lungs, BAL cells were evaluated by relative gene expression for markers of inflammation, cytokines, tight junction, and zinc-related genes at 0, 2, and 5 dpi (Fig. 5). All eight genes showed a time effect (*P* ≤ 0.01), with most targets being highly expressed at 2 dpi and then decreasing by 5 dpi. Treatment with ITM tended to increase (A) IL-8, (B) SerpinB2, and (C) MMP9 gene expression in BAL cells from ITMPRE compared to CON (*P* = 0.06). Gene expression of IL-8, SerpinB2, and MMP9 was intermediate in BAL cells from ITMPOST, compared to ITMPRE and CON. No other gene targets were affected by ITM treatment. Interleukin-8 is a proinflammatory cytokine that induces neutrophil and monocyte recruitment and activation. Previous reports have shown robust induction of IL-8 in bovine lungs infected with *M. haemolytica* [49, 61] and in in vitro cell culture models using primary bovine bronchial epithelial cells (BBEC) [62]. Exacerbated neutrophil accumulation and IL-8 induction is known to contribute the pathophysiology of severe *M. haemolytica* infection [48]. Interestingly, however, in calves experiencing severe disease, IL-8 expression is maintained at high levels rather than experiencing a peak and subsequent decline, as seen in the current study [61]. SerpinB2 is highly induced in macrophages and neutrophils in response to inflammation and infection [63]. N’jai also observed that *M. haemolytica* infection of BBEC caused a more than 20 fold increase in SerpinB2 over uninfected control cells [62]. While the importance of SerpinB2 in respiratory disease is not clear, some reports have linked SerpinB2 to M2 polarization of macrophages and regulation of inflammation [64, 65]. Matrix metalloproteinase 9 (MMP9) is a zinc-dependent collagenase. Neutrophils and activated macrophages release MMP9 to promote degradation of the extracellular matrix, enabling immune cell infiltration into the lungs [66]. We have previously observed upregulation of MMP9 in the lungs of calves with *M. haemolytica* infection, similar to that observed in the present study [49]. Both Zn and Se are intricately linked with MMP9 function [67], which may contribute to the increase in its expression in ITMPRE animals at 2 dpi. However, neutrophils and activated macrophages are a major source of MMP9 mRNA and protein. While it was not statistically significant, we observed a numerical increase in the accumulation of both populations in the lungs of ITMPRE and ITMPOST animals compared to CON. The increase in MMP9 gene expression may be attributed to the higher prevalence of these populations in the BAL at these timepoints after infection. Together, the gene expression data and flow cytometry results suggest that ITM treatment alters the inflammatory milieu in the lungs, which may be beneficial in the context of a respiratory bacterial infection.

## Conclusions

Together, our results suggest that ITM treatment improves the outcome of an experimental *M. haemolytica* infection. Cattle receiving ITM at receiving processing had the most improved clinical disease outcomes and TUS scores compared to untreated controls, although ITM treatment at the time of antibiotic treatment still gave some benefit. Importantly, the cattle in this study had adequate trace mineral levels at the time of enrollment, and the experimental challenge in the current study was relatively mild. Thus, a more stringent infection, or infection in trace mineral deficient animals, may demonstrate even greater benefit to receiving calves experiencing a health challenge. Treatment with ITM altered the inflammatory milieu in the lungs and peripheral blood of *M. haemolytica-*infected calves, promoting changes in monocyte subsets, γδ T cells, and NK cells that are important for host defense and maintenance of lung tissue integrity. Administration of ITM also resulted in improved capacity for proinflammatory cytokine secretion by circulating immune cells before infection, suggesting a readiness for disease challenge. Overall, the results support the use of ITM treatments to promote disease resilience in newly received beef calves.

## Data Availability

No datasets were generated or analyzed during the current study.
